# [Expression of Concern] 4-Hydroxybutenolide impairs cell migration, and invasion of human oral cancer SCC-4 cells via the inhibition of NF-κB and MAPK signaling pathways

**DOI:** 10.3892/ijo.2025.5795

**Published:** 2025-08-25

**Authors:** Fu-Shun Yu, Meng-Liang Lin, Shu-Chun Hsu, Chien-Chih Yu, Yi-Ping Huang, Yueh-Hsiung Kuo, Jing-Gung Chung

Int J Oncol 49: 579-588, 2016; DOI: 10.3892/ijo.2016.3537

Following the publication of the above paper, it was drawn to the Editor's attention by a concerned reader that, for the Transwell assay data shown in Fig. 2C on p. 582, the '24 h/1 *µ*M' and '24 h/2.5 *µ*M' data panels contained an overlapping section of cellular data; in addition, the '24 h/Control' and '48 h/Control' panels in Fig. 2E similarly contained an overlapping section of data, such that data which were intended to show the results from differently performed experiments appeared to have been derived from the same original sources. Upon analyzing the data independently in the Editorial Office, it came to light that the '0 h/1 *µ*M' and '12 h/2.5 *µ*M' data panels in Fig. 2A (showing the results of scratch-wound assay experiments) also contained an overlapping area, albeit the 12 h/2.5 *µ*M' data panel had been rotated through 180° relative to the '0 h/1 *µ*M' panel.

The authors were contacted by the Editorial Office to offer an explanation for these apparent anomalies in the presentation of the data in this paper, although up to this time, no response from them has been forthcoming. Owing to the fact that the Editorial Office has been made aware of potential issues surrounding the scientific integrity of this paper, we are issuing an Expression of Concern to notify readers of this potential problem while the Editorial Office conitnues to investigate this matter further.

